# Comprehensive Analysis of Pyroptosis-Related Long Noncoding RNA Immune Infiltration and Prediction of Prognosis in Patients with Colon Cancer

**DOI:** 10.1155/2022/2035808

**Published:** 2022-01-18

**Authors:** Li Liu, Wenzheng Chen, Yebei Li, Pengcheng Fu, Yi Cao, Zhengrong Li, Jianbo Xiong, Zhigang Jie

**Affiliations:** ^1^Department of Gastrointestinal Surgery, The First Affiliated Hospital of Nanchang University, Nanchang, China; ^2^The Second Affiliated Hospital of Nanchang University, Nanchang, China

## Abstract

Colon cancer (CC) is one of the most prevalent malignant tumours of the alimentary canal. It is unclear whether pyroptosis-related lncRNA expression is correlated with CC prognosis. We discovered 20 pyroptosis-related lncRNAs that were expressed differently in CC and normal colon tissues in our investigation. Based on differentially expressed genes (DEGs), we grouped all CC patients into two categories (Clusters 1 and 2). Cluster 1 was shown to be connected with a higher overall survival rate, upregulated expression of immune checkpoints, higher immunoscores, higher estimated scores, and immune cell infiltration. Using data from the Cancer Genome Atlas (TCGA), to create a multigene signature, the predictive significance of each lncRNA linked with pyroptosis for survival was assessed. A 9-lncRNA signature was established using the least absolute shrinkage and selection operator (LASSO) Cox regression method, and all CC patients in the TCGA cohort were classified into low-risk or high-risk groups. The low-risk CC patients had a much greater chance of survival than those in the high-risk group. The risk score is an independent prognostic indicator for predicting survival. In addition, risk characteristics are linked to immune characteristics. In summary, pyroptosis-related lncRNAs can be used to predict CC prognosis and participate in tumour immunity.

## 1. Introduction

Colon cancer (CC) is one of the most frequent tumours globally, accounting for 6% of global tumour incidence in 2020, and the morbidity and mortality rate ranked fifth among cancers in 2020 [1]. The prevalence of CC has reduced marginally in the last few years, but the increase in the prevalence rate among young people has become more visible [2]. However, the pathophysiology of CC is not completely known, and the diagnosis and treatment of CC are still controversial [3–5]. There are still many questions waiting for us to discover and solve.

Pyroptosis is a type of programmed cell death (PCD) and is defined by the morphology of inflammatory cell death. It was first observed in 1992 [6], and then Boise and Collins named it pyroptosis in 2000 [7]. The Gasdermin family is what distinguishes it. The cells enlarge, dissolve, and release proinflammatory cytokines, such as IL-1*β* and IL-18, after pores mediated by the Gasdermin family are formed. Its occurrence mode includes classical and nonclassical pathways [8–13]. The thermophilic cells create a significant number of vesicles first, as seen under the electron microscope. When these vesicles are formed, holes are formed in the cell membrane. The holes burst, and the contents flow out [14]. Pyroptosis has been implicated in tumour development, mortality, and the tumour immune microenvironment in numerous studies [15]. Increasing data are proving that pyroptosis is important in the onset and development of cancers [16–19].

Through different biological functions, long noncoding RNAs (lncRNAs) play a role in CC pathogenesis regulation [20–22]. For example, lncRNA H19 has the capability to enhance the development of CC cells [23]. The presence of KCNQ1OT1 is likewise associated with the growth of CC [24]. T-UCRs, CCAT1, and many other lncRNAs have also been found to be linked to the development of CC [25–27]. In addition, lncRNAs are related to immune cells and the tumour immune microenvironment [28, 29]. Tumour immunity is thought to have a significant influence on tumour development [30]. The immunosuppressive microenvironment can be induced by lncRNAs in various ways, to control tumour escape from immune surveillance and promote tumour metastasis and drug resistance. Ren et al. found that the tumour suppressor lncRNA ADAMTS9-AS2 triggers NLRP3-mediated thermophilic cell demise by sponging miR-223-3p, increasing the sensitivity of GC cells to cisplatin [31]. Other studies also suggest that lncRNAs may be associated with pyroptosis and malignant tumours [32, 33].

Nevertheless, it is unclear whether lncRNAs linked to pyroptosis are associated with the prognosis of CC. In our research, TCGA database data was used to construct and verify lncRNA prognostic markers associated with pyroptosis; and their potential mechanism in CC was discussed. In this study, lncRNA prognostic markers related to pyroptosis were constructed and verified for the first time. We analysed the tumour microenvironment, immune cell infiltration, immune checkpoint inhibitors, functional enrichment, chemotherapy sensitivity, and so on. This research might help us better grasp the link between pyroptosis-related lncRNA expression and CC and predict prognosis and treatment outcomes in individuals with CC.

## 2. Materials and Methods

### 2.1. Data Collection

The TCGA database was used to gather the mRNA expression profiles of 473 COAD patients as same as their clinical parameters (age, survival status, and grade, for instance) [34] (https://portal.gdc.cancer.gov/repository) on September 22, 2021. We also obtained 41 normal colonic tissue samples from the TCGA database. Data for 51 pyroptosis-related genes were procured from the GSEA website (https://www.gsea-msigdb.org/gsea) and prior reviews [12, 35–38]. Before being compared, the expression data were standardized to fragment per kilobase million (FPKM) values [39].

### 2.2. Identification of Differentially Expressed Genes (DEGs) of Pyroptosis and Pyroptosis-Related lncRNAs

To identify differentially expressed genes related to pyroptosis, we employed the “limma” software program, and the standard was FDR<0.05, | logFC | > 1. The DEGs are noted as follows: ^*∗*^ if *P* < 0.05, ^*∗∗*^ if *P* < 0.01, and ^*∗∗∗*^ if *P* < 0.001. With p < 0.001 and |Pearson R| > 0.4, the “limma” package can also be used to build a coexpression network comprising differentially expressed genes related to pyroptosis and lncRNAs, and the “igraph” package was employed to map the coexpression network. To explore prognostic pyroptosis-related lncRNAs, univariate Cox regression analysis was employed.

### 2.3. Consensus Clustering and Immune Correlation Analysis for Prognostic Pyroptosis-Related lncRNAs

All CC data were divided into subgroups based on the expression of prognostic pyroptosis-related lncRNAs using the “ConsensusClusterPlus” package. Then, we analysed the difference in survival probability, expression, and coexpression of immune checkpoint inhibitors (PD-1, PD-L1, and CTLA-4), immune cell infiltration, and immune cell-related score in all clusters. Using the ESTIMATE algorithm to figure immuneScore and stromalScore with the “estimate” package. Gene set enrichment analyses (GSEAs) were also carried out.

### 2.4. Creation and Verification of the Pyroptosis-Related lncRNA Prognostic Model

The prognostic signature of 9 pyroptosis-related lncRNA was created using least absolute shrinkage and selection operator (LASSO) regression analysis to evaluate their fatidic significance [40] (risk score = Ʃ (Exp [lncRNA] × coef [lncRNA])). Exp (lncRNA) is the corresponding expression of the included lncRNAs, and coef (lncRNA) represents the regression coefficient. We separated all samples into training and testing groups at a 1 : 1 ratio and then into high-risk and low-risk groups based on the median risk score in both the training and the testing groups.

Survival analysis, receiver operating characteristic (ROC) curve [41], the areas under the time-dependent ROC curve (AUCs), independent prognostic analysis based on Cox regression [42], and nomogram [43] have also been structured. To test the model's value, we examined the link between risk score and survival probability in different clinicopathological features. We also reviewed the association between tumour immunity, clinicopathological characteristics, risk scores, and immune checkpoint expression, which is momentous in tumour [44]. The immune cell enrichment data come from the TIMER2.0 database [45] (https://timer.cistrome.org/).

### 2.5. Sensitivity Analysis of Chemotherapeutic Drugs

Using the “pRRophetic” package, we predicted the link between chemotherapeutic drug sensitivity and risk score. This prediction may be good for therapy. Three chemotherapeutic drugs (cisplatin, docetaxel, and paclitaxel) were included in our study. To contrast the discrepancy, the Wilcoxon signed-rank test was employed.

### 2.6. Statistical Analysis

For statistical analysis and outcome display, *R* software (version 4.1.0) was utilized. The Benjamini–Hochberg method was utilized to authenticate differently expressions. The Mann–Whitney *U* test was utilized to detect the mRNA level of pyroptosis-related lncRNAs. Student's *t*-test was utilized to determine the differences between the two groupings. The classification variables in the training and testing tests were contrasted using the chi-square test. The link between subtypes, clinicopathological factors, risk score, immune check inhibitors, and immune infiltration levels was assessed using the Pearson correlation test. The Kaplan–Meier method [46] with a two-sided log-rank test was employed for survival analysis.

## 3. Result

### 3.1. Differentially Expressed Pyroptosis-Related Genes

**Figure 1:**
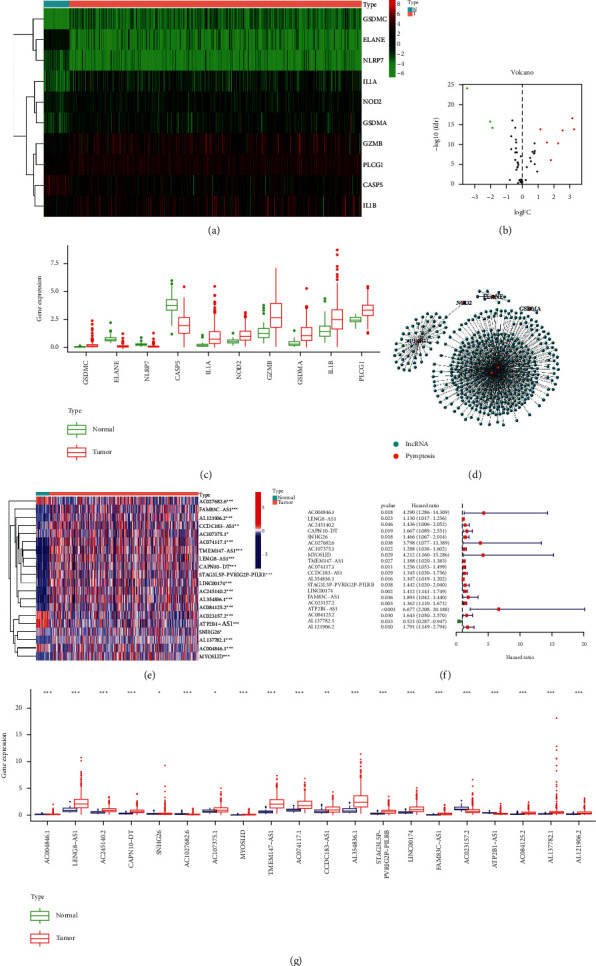
Differentially expressed genes of pyroptosis and pyroptosis-related lncRNAs in colon tumour and adjacent normal tissues. (a) Heatmap shows differentially expressed genes of pyroptosis, (b) volcano plot shows differentially expressed genes of pyroptosis, (c) boxplot shows differentially expressed genes of pyroptosis, (d) gene coexpression network map of pyroptosis genes and lncRNAs, (e) heatmap shows differentially expressed genes of pyroptosis-related lncRNAs, (f) forest plot shows prognostic related genes of pyroptosis-related lncRNAs, and (g) boxplot shows differentially expressed genes of pyroptosis-related lncRNAs. ^*∗*^*P* < 0.05, ^*∗∗*^*P* < 0.01, and ^*∗∗∗*^*P* < 0.001.

The expression levels of 51 pyroptosis-related genes were examined in TCGA data from 41 normal and 473 tumour samples, and 10 DEGs were discovered (FDR <0.05, | logFC | > 1). Three genes (ELANE, NLRP7, and CASP5) were downregulated, whereas seven others (GSDMC, IL1A, NOD2, GZMB, GSDMA, IL1B, and PLCG1) were overrepresented in the tumour group. These genes' RNA levels are displayed (Figures 1(a)–1(c)).

### 3.2. Pyroptosis-Related lncRNAs in Colon Cancer

We confirmed 462 lncRNAs with a coexpression relationship in CC data (corFilter = 0.4, pvalueFilter = 0.001) (Figure 1(d)). Twenty differentially expressed prognosis related lncRNAs were explored by univariate Cox analysis: AC004846.1, LENG8-AS1, AC245140.2, CAPN10-DT, SNHG26, AC027682.6, AC107375.1, MYOSLID, TMEM147-AS1, AC074117.1, CCDC183-AS1, AL354836.1, STAG3L5P-PVRIG2P-PILRB, LINC00174, FAM83C-AS1, AC023157.2, ATP2B1-AS1, AC084125.2, AL137782.1, and AL121906.2. These pyroptosis-related lncRNAs have significant expression differences in CC (Figure 1(e)). Five of these lncRNAs (AC004846.1, SNHG26, AC027682.6, AC023157.2, and ATP2B1-AS1) were downregulated, whereas the others were upregulated, compared with normal tissues (LENG8-AS1, AC245140.2, CAPN10-DT, AC107375.1, MYOSLID, TMEM147-AS1, AC074117.1, CCDC183-AS1, AL354836.1, STAG3L5P-PVRIG2P-PILRB, LINC00174, FAM83C-AS1, AC084125.2, AL137782.1, and AL121906.2) (Figures 1(f) and 1(g)).

### 3.3. Pyroptosis-Related lncRNAs: Consensus Clustering

**Figure 2:**
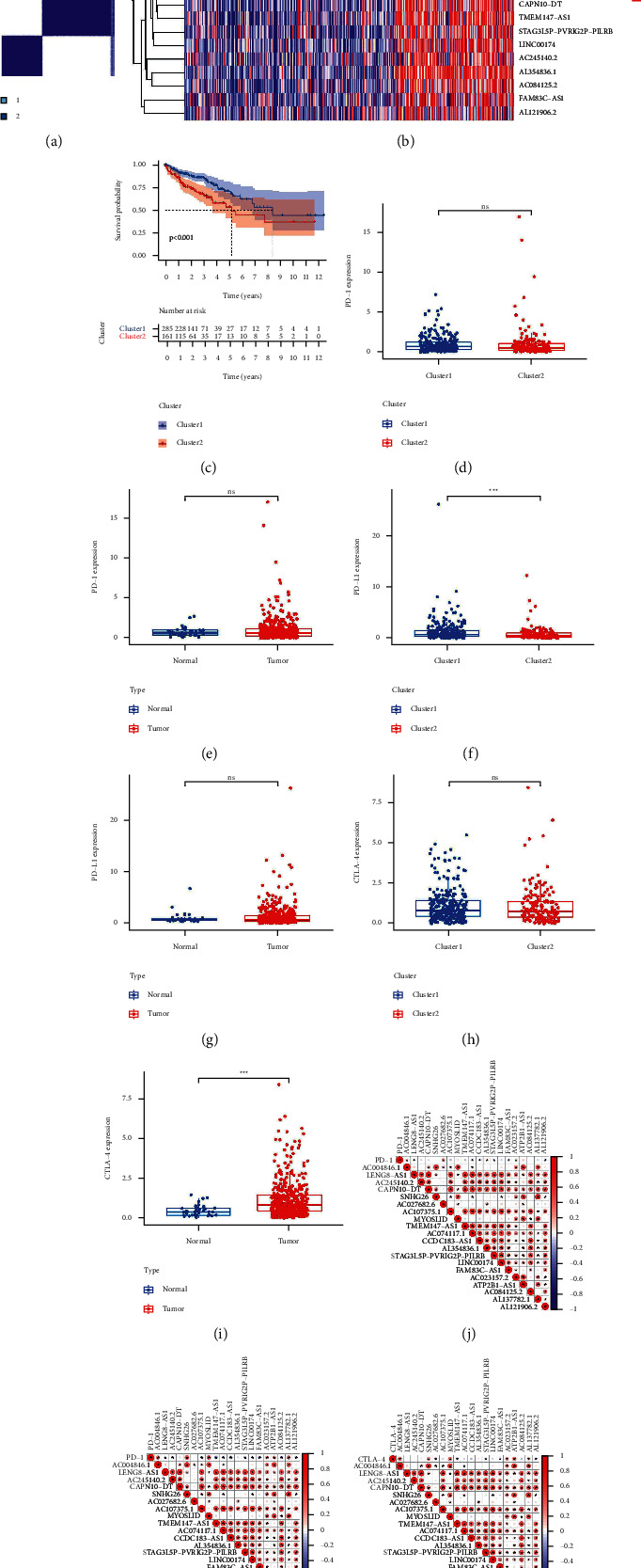
Clinical and pathological characteristics, overall survival and association of immune checkpoint inhibitors of colon cancer patients in Clusters 1 and 2. (a) Consensus clustering matrix for *k* = 2, (b) heatmap and clinicopathologic features of Clusters 1 and 2, (c) Kaplan–Meier curves of overall survival (OS) of Clusters 1 and 2, (d) PD-1 expression levels of Clusters 1 and 2, (e) PD-1 expression levels in normal sample and tumour sample, (f) PD-L1 expression levels of Clusters 1 and 2, (g) PD-L1 expression levels in normal and tumour sample, (h) CTLA-4 expression levels of Clusters 1 and 2, (i) CTLA-4 expression levels in normal and tumour sample, (j) correlation between PD-1 expression level and differential expression of pyroptosis-related lncRNAs, (k) correlation between PD-L1 expression level and differential expression of pyroptosis-related lncRNAs, and (l) correlation between CTLA-4 expression level and differential expression of pyroptosis-related lncRNAs. ^*∗*^*P* < 0.05, ^*∗∗*^*P* < 0.01, and ^*∗∗∗*^*P* < 0.001.

To investigate the association between COAD subtypes and the expression of the 20 pyroptosis-related differentially expressed lncRNAs, consensus clustering analyses [47] were used on all 473 COAD samples in TCGA. The tumour samples were divided into clusters via the “ConsensusClusterPlus” *R* package. We discovered that when the clustering variable (*k*) was set to 2, the highest intragroup correlations were found, whereas the lowest intergroup correlations were found (Supplementary Figure 1 and Figure 2(a)). In a heatmap displaying gene expression and clinical characteristics, such as tumour stage and age, we discovered that the two clusters differed considerably in terms of regional lymph node (N), metastasis (M), and tumour stage (Figure 2(b)). Overall survival time (OS) showed significant differences among clusters, and Cluster 1 had a higher chance of surviving than Cluster 2 (Figure 2(c)).

### 3.4. The Correlation between Consensus Clustering for Pyroptosis-Related lncRNAs and Immune Checkpoint Inhibitors and Immune Cell Infiltration

**Figure 3:**
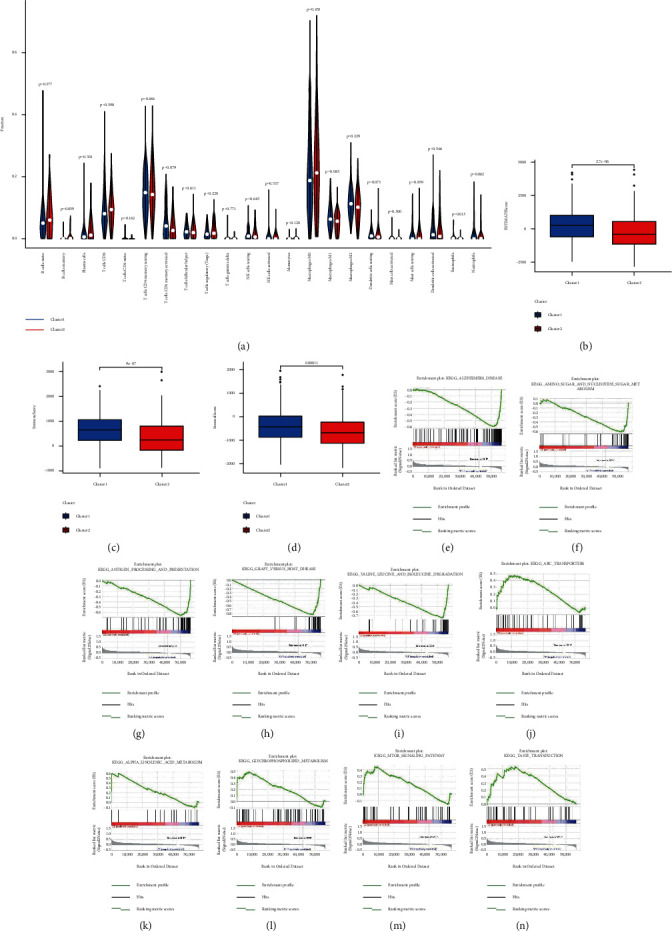
The difference between immune cell infiltration levels and scores in two clusters and distinct pathways enriched in Clusters 1 and 2. (a) The infiltration of 22 immune cell types in Clusters 1 and 2, (b) ESTMATE score in Clusters1 and 2, (c) immune score in Clusters 1 and 2, (d) stromal score in Clusters 1 and 2, ((e)–(i)) top five pathways enriched in Cluster 1, (e) Alzheimer's disease, (f) amino sugar and nucleotide sugar metabolism, (g) antigen processing and presentation, (h) graft versus host disease, (i) valine, leucine, and isoleucine degradation, ((j)–(n)) top five pathways enriched in Cluster 2, (j) ABC transporters, (k) alpha-linolenic acid metabolism, (l) glycerophospholipid metabolism, (m) motor signalling pathway, and (n) taste transduction.

The relationship between consensus clustering and tumour immunity is one of the points we researched. We examined the expression differences of immune checkpoints (PD-1, PD-L1, and CTLA-4) in the two clusters and examined the differences between tumour and normal tissues. The findings revealed that PD-L1 expression was higher in Cluster 1 than in Cluster 2, whereas the expression of CTLA-4 was higher in tumour than in normal (Figures 2(d)–2(i)). The expression levels of 20 pyroptosis-related lncRNAs and immunological checkpoints were also investigated and showed a significant correlation. The expression level of PD-1 was related to AC004846.1, AC027682.6, AC023157.2, LENG8-AS1, AC074117.1, CCDC183-AS1, AL354836.1, STAG3L5P-PVRIG2P-PILRB, FAM83C-AS1, and AL121906.2 (Figure 2(j)). The expression level of PD-L1 was related to AC004846.1, LENG8-AS1, AC245140.2, SNHG26, AC107375.1, MYOSLID, TMEM147-AS1, AC074117.1, CCDC183-AS1, STAG3L5P-PVRIG2P-PILRB, LINC00174, FAM83C-AS1, AC023157.2, ATP2B1-AS1, AC084125.2, AL137782.1, and AL121906.2 (Figure 2(k)). The expression level of CTLA-4 was related to AC004846.1, SNHG26, AC027682.6, MYOSLID, TMEM147-AS1, STAG3L5P-PVRIG2P-PILRB, FAM83C-AS1, AC023157.2, ATP2B1-AS1, AL137782.1, and AL121906.2 (Figure 2(l)). The differences of the infiltration fractions of 22 immune cells (plasma cells, eosinophils, macrophages M0, macrophages M1, macrophages M2, monocytes, mast cells activated, mast cells resting, neutrophils, NK cells activated, NK cells resting, T cells CD4 memory activated, T cells CD4 memory resting, T cells CD4 naive, T cells CD8, T cells follicular helper, T cells gamma delta, T cells regulatory (Tregs), B cells memory, B cells naive, dendritic cells activated, and dendritic cells resting) in two clusters were explored, but we discovered that the clinical features of the two clusters were nearly identical (Figure 3(a)). However, there was a statistically obvious difference in the ESTMATE score, immune score, and stromal score between the two clusters (Figures 3(b)–3(d)). Cluster 1 had a larger level of immunological infiltration than Cluster 2, according to the findings.

### 3.5. Enrichment Analysis of Each Colon Cancer Subtype

The possible regulatory mechanisms that led to differences between the two groups were elucidated using GSEA. Some cancer- and metabolism-related pathways were enriched by gene set enrichment analyses (Figures 3(e)–3(n)), including amino sugar and nucleotide sugar metabolism, antigen processing and presentation, valine, leucine, and isoleucine degradation, which were closely associated with Cluster 1, as well as alpha-linolenic acid metabolism, glycerophospholipid metabolism, and the motor signalling pathway, which were closely related to Cluster 2.

### 3.6. Construction of the Prognostic Model

**Figure 4:**
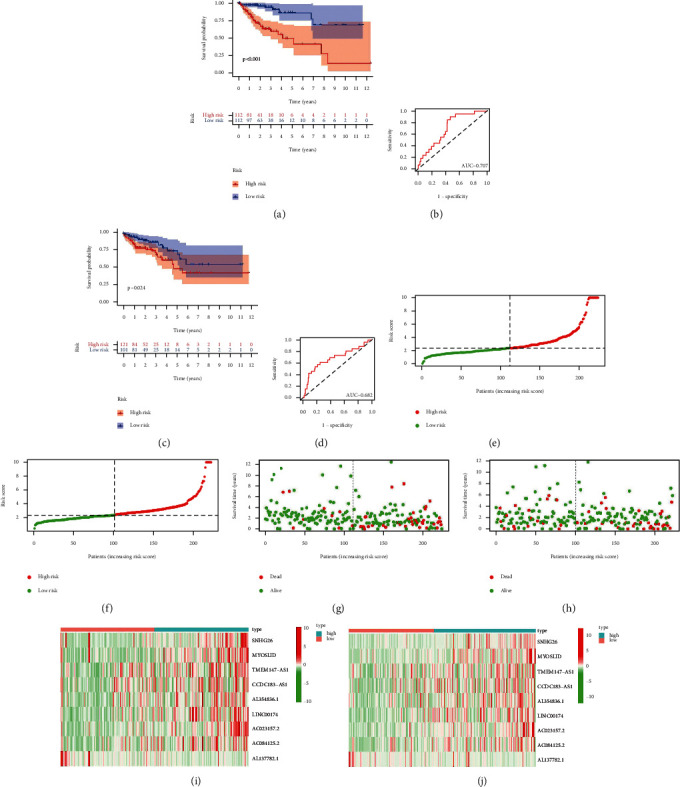
Construction and validation of prognostic model for pyroptosis-related lncRNAs in CC. (a) Kaplan–Meier curve for OS in training group, (b) ROC curve in training group, (c) Kaplan–Meier curve for OS in testing group, (d) ROC curve in testing group, (e) risk score distribution in training group, (f) risk score distribution in testing group, (g) OS status in training group, (h) OS status in testing group, (i) heatmap in training group, and (j) heatmap in testing group.

We divided all 473 COAD samples into two cohorts on average, with one group as the training cohort and the other as the testing cohort. We built a LASSO regression model based on univariate Cox regression analysis to predict the prognosis of COAD patients (Supplementary Figure 2). Nine lncRNAs (SNHG26, MYOSLID, TMEM147-AS1, CCDC183-AS1, AL354836.1, LINC00174, AC023157.2, AC084125.2, and AL137782.1) were identified for further analysis, and the risk score was calculated using the following formula: risk score = (0.6661 *∗* SNHG26 exp.) + (2.8228 *∗* MYOSLID exp.) + (0.0290 *∗* TMEM147-AS1 exp.) + (0.0793 *∗* CCDC183-AS1 exp.) + (0.0670 *∗* AL354836.1 exp.) + (0.0725 *∗* LINC00174 exp.) + (0.3680 *∗* AC023157.2 exp.) + (0.2130 *∗* AC084125.2 exp.) + (−0.5407 *∗* AL137782.1 exp.). We separated 473 samples into high-risk and low-risk groups based on the median value of the risk score in the training and testing groups. Survival analysis confirmed that the low-risk patients had a better prognosis than the high-risk (Figures 4(a)–4(c)). AUCs of the training and testing cohort were 0.707 and 0.682, indicating that the risk scores generated using the 9 pyroptosis-related lncRNA signatures had better prediction performance (Figures 4(b) and 4(d)). A heatmap of clinicopathological features and risk groups is also presented (Figures 4(i) and 4(j)).

### 3.7. Independent Prognostic Analysis and Nomogram

**Figure 5:**
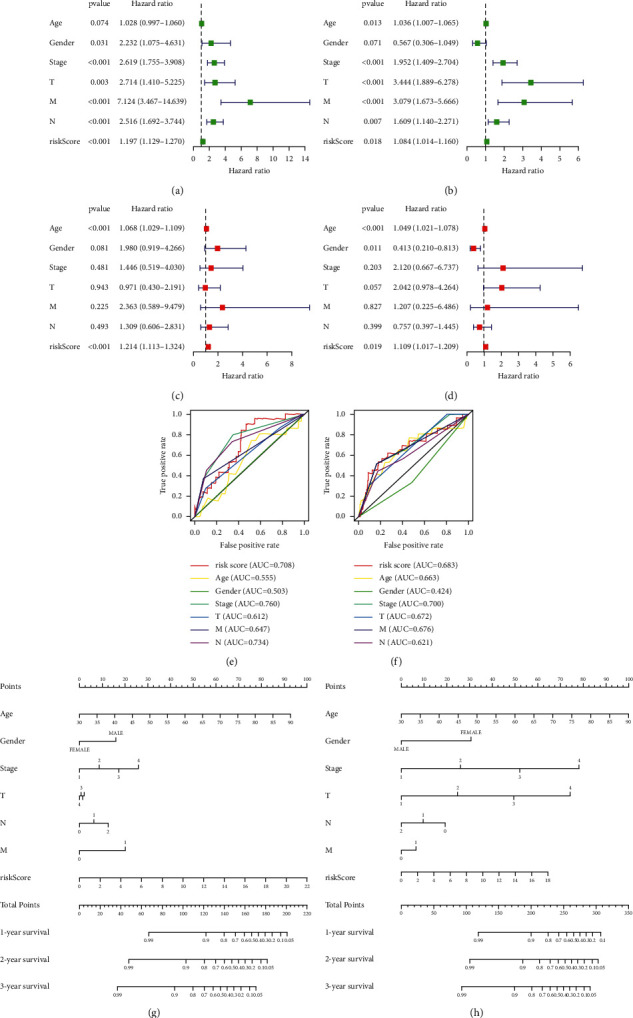
Independent prognostic analysis and nomogram plot of training and testing group. (a) Univariate independent prognostic analysis in training group, (b) univariate independent prognostic analysis in testing group, (c) multivariate independent prognostic analysis in training group, (d) multivariate independent prognostic analysis in testing group, (e) clinical factors ROC curve in training group, (f) clinical factors ROC curve in testing group, (g) nomogram based on clinical factors and risk score in training group, and (h) nomogram based on clinical factors and risk score in testing group.

We performed univariate independent prognostic analysis and multivariate independent prognostic analysis forest maps utilizing clinical data from the TCGA database to determine whether the risk score could be used as an independent prognostic factor. The results showed that stage, *T* stage, N stage, *M* stage, and risk score were independent predictive factors in both cohorts, according to the univariate Cox regression analysis. The risk score was a prognostic factor for COAD patients in both cohorts, according to the multivariate analysis (Figures 5(a)–5(d)). The ROC curve and AUC value of clinically related factors are shown (Figures 5(e) and 5(f)). A nomogram was created to predict patient survival rates in both cohorts (Figures 5(g) and 5(h)).

### 3.8. Risk Score and Clinicopathological Characteristics

**Figure 6:**
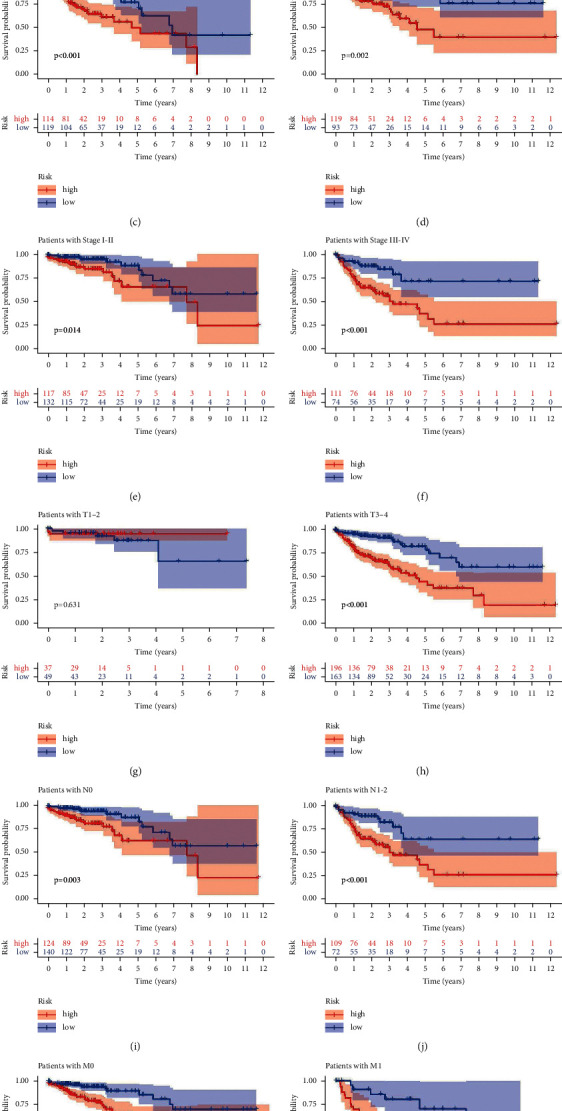
Kaplan–Meier survival subgroup analysis for the prognostic signature of 9 pyroptosis-related lncRNAs stratified by clinical characteristics. ((a), (b)) Patients aged ≤65 years and >65 years, ((c), (d)) men and women, ((e), (f)) stages I-II and III-IV, ((g), (h)) T1-2 and T3-4, ((i), (j)) N0 and N1-2, and ((k), (l)) M0 and M1.

**Figure 7:**
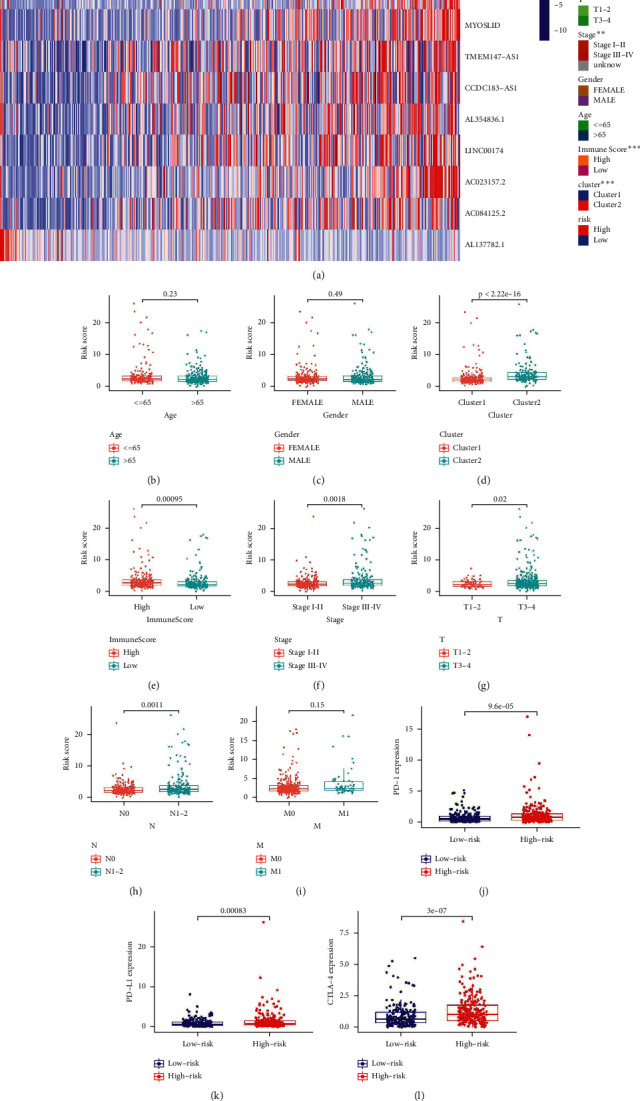
Heatmap and boxplots for differential clinicopathological features of high- and low-risk scores group and the difference of target gene expression between the two groups. (a) Heatmap, (b) boxplot of the differences in risk scores between age ≤65 and age >65, (c) boxplot of the differences in risk scores between male and female, (d) boxplot of the differences in risk scores between Cluster 1 and Cluster 2, (e) boxplot of the differences in risk scores between high expression and low expression, (f) boxplot of the differences in risk scores between stage I-II and stage III-IV, (g) boxplot of the differences in risk scores between T1-2 and T3-4, (h) boxplot of the differences in risk scores between N0 and N1-2, (i) boxplot of the differences in risk scores between M0 and M1, (j) differential expression of PD-1 between high- and low-risk groups, (k) differential expression of PD-L1 between high- and low-risk groups, and (l) differential expression of CTLA-4 between high- and low-risk groups.

In diverse clinicopathological features, we surveyed the relationship between risk score and survival probability, including patients age ≤65 years, patients age >65 years, male patients, female patients, patients with stage I-II, patients with stage III-IV, patients with T1-2, patients with T3-4, patients with N0, patients with N1-2, patients with M0, and patients with M1. Except for patients with T1-2 disease, the prognosis for the low-risk group was better than the high-risk group according to the findings (Figure 6). This outcome also confirmed the reliability of the model. The heatmap and boxplot demonstrated that high-risk patients were significantly correlated with N classification, stage, immune score, and cluster (Figures 7(a)–7(i)).

### 3.9. Correlation Analysis of Immunity and Sensitivity Analysis of Chemotherapeutic Drugs

**Figure 8:**
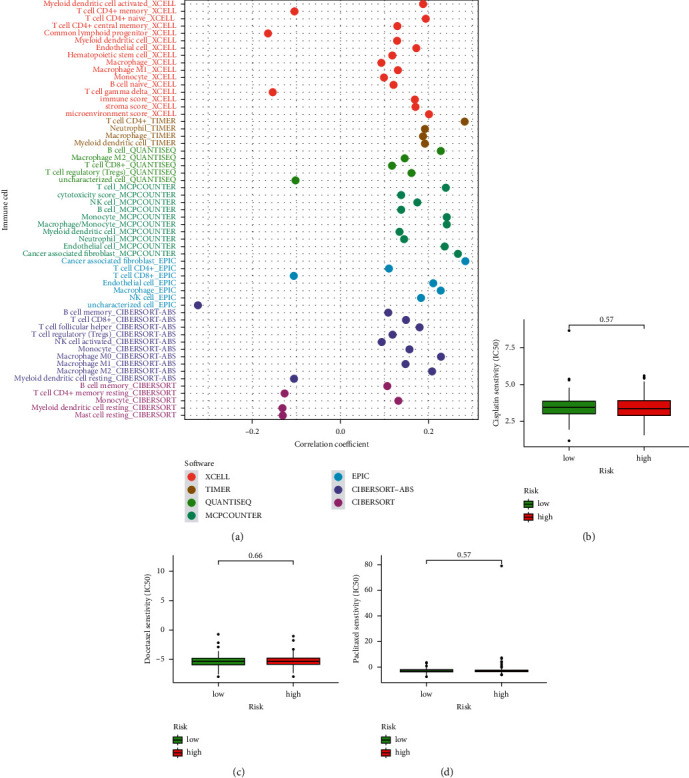
Correlation analysis of immune cells in different software and the difference of drug sensitivity in high-risk groups and low-risk groups. (a) Correlation analysis of immune cells in various software, (b) cisplatin sensitivity in high-risk group and low-risk group, (c) docetaxel sensitivity in high-risk group and low-risk group, and (d) paclitaxel sensitivity in high-risk group and low-risk group.

Some researchers have pointed out that the immune microenvironment is related to pyroptosis-related genes [15]. Immune checkpoints (PD-1, PD-L1, and CTLA-4) were evaluated differently in two groups. High-risk groups had higher expression of three immunological checkpoints than low-risk groups (Figures 7(j)–7(l)). Based on data from the TIMER2.0 database, we created a bubble chart to identify the association between immune cells and the risk score. The findings of various software predictions revealed that immune cells and risk score had a favourable relationship (Figure 8(a)). We wanted to see if there was a link between the risk score and chemotherapeutic efficacy in treating CC. We identified the association between risk scores and the sensitivity of three chemotherapeutic drugs (cisplatin, docetaxel, and paclitaxel). However, between the two groups, there was no discernible variation in drug sensitivity among the three types of chemotherapeutic medicines (Figures 8(b)–8(g)).

## 4. Discussion

One of the most prevalent malignant tumours of the alimentary canal is colon cancer (CC). CC normally develops at the intersection of the rectum and sigmoid colons, and CC is the third most frequent kind of tumour in the digestive tract [48]. In the treatment of CC, many molecules related to prognosis have been found, and clinical treatment is also increasing, which benefits increasing number of patients [49–51]. LncRNAs and pyroptosis are significant factors in the emergence and progression of malignancies [16–21, 24–27, 52,53]. The function of pyroptosis-related lncRNAs in several cancers has been verified by researchers [31–33]. However, there is no related research report on CC. Therefore, the status and mechanisms of pyroptosis-related lncRNAs in CC need to be explored for future treatment.

Pyroptosis is a sign of PCD that occurs in cells infected by pathogens and causes inflammation in the body [54]. Pyroptosis, in contrast to other kinds of cell death, is a highly inflammatory form of PCD that is entirely driven by caspase-1 cleavage. Not only does the conversion of precursor caspase-1 to cleaved caspase-1 result in the formation of cell membrane pores, a loss of membrane integrity, and the release of intracellular inflammatory substances, but it also promotes the cleavage of precursors IL-18 and IL-1*β* into mature proinflammatory IL-18 and IL-1*β*, aggravating the cell inflammation process. Pyroptosis also has a dual function in the growth and therapy of malignancies [38]. On the one hand, pyroptosis releases large number of inflammatory factors to stimulate normal cells, which causes them to transform into tumour cells. On the other hand, inducing tumour cell pyroptosis may become a novel therapeutic target [12]. It is unclear how pyroptosis-related genes interact in CC or whether they are linked to patient prognosis. In our research, for the first time, pyroptosis-related lncRNAs were separated into subgroups to develop prognostic indicators, and a thorough examination of the link among the tumour microenvironment, immune cell infiltration, immunological checkpoints, and pyroptosis-related lncRNAs to advise therapy was conducted.

We gathered genes associated with pyroptosis from the literature and the GSEA website, screened DEGs in CC, and examined the coexpression of pyroptosis-related lncRNAs, resulting in the discovery of 20 lncRNAs linked to prognosis with differential expression. In CC, we discovered and confirmed two pyroptosis-related lncRNA subgroups. Cluster 2 had a worse overall survival rate than Cluster 1, and the expression of pyroptosis-related prognostic lncRNAs in Cluster 1 was typically lower than that in Cluster 2. The tumour microenvironment has a significant regulatory function in tumour growth and heterogeneity, influencing patient prognosis and curative outcomes [55, 56]. We found that there was an obvious difference in tumour microenvironments between the two subtypes, and Cluster 1 had higher immune, stromal, and estimated scores. Cluster 1 had a much larger quantity of eosinophils than Cluster 2. Immunotherapy is currently considered to be an effective method of tumour treatment. In Cluster 1, the PD-L1 expression levels were higher. These findings indicate that Cluster 1 had more immunological infiltration than Cluster 2 and that Cluster 1 patients may have a good curative effect after receiving immunotherapy. Interestingly, these conclusions are consistent; that is, CC patients with a better overall survival rate had higher immune scores, and immune checkpoint expression was higher. Our research was focused on these elements that directly cause tumour cell death or alter the tumour immune microenvironment, which may provide a reference for treatment.

It is not clear how pyroptosis-related lncRNAs interact with each other in CC and whether they are related to prognosis. Our study found a signature featuring 9-lncRNAs related to pyroptosis (SNHG26, MYOSLID, TMEM147-AS1, CCDC183-AS1, AL354836.1, LINC00174, AC023157.2, AC084125.2, and AL137782.1) and revealed that it has the ability to anticipate the prognosis of people with CC. Wang Y. and Tong H. showed that SNHG26 is closely associated with the tumour, immune microenvironment, CC, and bladder cancer [57, 58]. MYOSLID promotes the progression of osteosarcoma through the miR-1286/RAB13 axis [59], and MYOSLID plays a key role in stomach neoplasm through the miR-29c-3p-mcl-1 axis [60], but it has not been reported in CC. CCDC183-AS1 enhances hepatocellular carcinoma progression by regulating SKP1 expression via MIR-589-5P [61]. The miR-3127-5p/E2F7 axis increases CC cell proliferation and migration, and LINC00174 plays a role in this process [62]. Other lncRNAs we extracted have not yet been reported in CC. Based on TCGA lncRNA expression data as well as clinical data, we constructed a prognostic model. Clinicopathological analysis and survival analysis showed this model has better sensitivity in predicting prognosis. The independent prognostic analysis we constructed also shows that it is credible to use these signatures as independent factors for evaluating prognosis. The nomogram used to predict the clinical prognosis was also constructed. The analysis of the signature and tumour immunity, immune cell level, and immune checkpoint also supported the considerable role of the signature in CC. These findings implied that the prognosis for CC patients in the high-risk group was poorer than for those in the low-risk group and that the signature of nine pyroptosis-related lncRNAs played a considerable role in determining the prognosis of CC.

First, our study conducted cluster analysis of pyroptosis-related lncRNAs in CC for the first time. Second, this paper examined the link between pyroptosis-related lncRNAs and prognostic markers in the tumour microenvironment as well as immune cell infiltration for the first time, which provided a new idea for the predictive function of pyroptosis-related lncRNA markers in immunotherapy. Third, this paper researched the relationship between the characteristics of pyroptosis-related lncRNAs and immune checkpoint expression as well as chemosensitivity for the first time. This may be helpful to clinical treatment. Our research does, however, have certain limitations. First, our research is based on TCGA data, which might lead to bias. If we comprehensively analyse the data from other sources, it may lead to different results. Second, we did not conduct experiments to prove the differences in the levels of molecular transcription and expression, which undoubtedly reduces its credibility. Finally, we do not have enough clinical follow-up data to back up our prognostic model.

## 5. Conclusion

In this research, we evaluated the value of pyroptosis-related lncRNAs in predicting prognosis, the tumour microenvironment's and immune cell infiltration's roles, potential regulatory mechanisms of pyroptosis-related lncRNAs, and the correlation between immune checkpoints and chemosensitivity of CC. The identifying features of nine lncRNAs related to pyroptosis properly predict the prognosis of patients with CC, which might aid in the development of customized treatment regimens and provide new insight into advanced therapies.

## Data Availability

All data sources are obtained from the Cancer Genome Atlas (TCGA) (https://portal.gdc.cancer.gov/repository) and TIMER2.0 database (https://timer.cistrome.org/) and processed by *R* software (version 4.1.0.).

## Supplementary Materials

Supplementary Materials Table S1. Pyroptosis-related genes. Table S2. Pyroptosis-related genes difference. Table S3. Pyroptosis-related lncRNA geneCoef. Table S4. Immune correlation result. Supplementary Figure 1. Consensus clustering of the tumourous cohort from TCGA based on differentially expressed pyroptosis-related lncRNAs. (A) Consensus clustering distribution function (CDF) for *k* = 2 to 9, (B) area under CDF curve increment for *k* = 2 to 9, and (C) tracking plot for *k* = 2 to 9. Supplementary Figure 2. LASSO regression analysis was used to identify nine pyroptosis-related lncRNAs. (A) The pyroptosis-related lncRNAs' LASSO coefficient profiles and (B) the LASSO regression model demonstrated partial likelihood deviance of various numbers of variables.
