# Supplementary material for: Comprehensive Analysis of Pyroptosis-Related Long Noncoding RNA Immune Infiltration and Prediction of Prognosis in Patients with Colon Cancer
Source: J Oncol. 2022 Jan 18;2022:2035808. doi: 10.1155/2022/2035808 (PMC8789477; doi:10.1155/2022/2035808)
Supplement: Supplementary Materials — Table S1. Pyroptosis-related genes. Table S2. Pyroptosis-related genes difference. Table S3. Pyroptosis-related lncRNA geneCoef. Table S4. Immune correlation result. Supplementary Figure 1. Consensus clustering of the tumourous cohort from TCGA based on differentially expressed pyroptosis-related lncRNAs. (A) Consensus clustering distribution function (CDF) for k = 2 to 9, (B) area under CDF curve increment for k = 2 to 9, and (C) tracking plot for k = 2 to 9. Supplementary Figure 2. LASSO regression analysis was used to identify nine pyroptosis-related lncRNAs. (A) The pyroptosis-related lncRNAs' LASSO coefficient profiles and (B) the LASSO regression model demonstrated partial likelihood deviance of various numbers of variables. [file 2035808.f1.docx]

**Table S1. Pyroptosis related genes.**

| **Genes** | **Description** |
| --- | --- |
| AIM2 | absent in melanoma 2 |
| BAK1 | BCL2 antagonist/killer 1 |
| BAX | BCL2 associated X, apoptosis regulator |
| CASP1 | caspase 1 |
| CASP3 | caspase 3 |
| CASP4 | caspase 4 |
| CASP5 | caspase 5 |
| CASP6 | caspase 6 |
| CASP8 | caspase 8 |
| CASP9 | caspase 9 |
| CHMP2A | charged multivesicular body protein 2A |
| CHMP2B | charged multivesicular body protein 2B |
| CHMP3 | charged multivesicular body protein 3 |
| CHMP4A | charged multivesicular body protein 4A |
| CHMP4B | charged multivesicular body protein 4B |
| CHMP4C | charged multivesicular body protein 4C |
| CHMP6 | charged multivesicular body protein 6 |
| CHMP7 | charged multivesicular body protein 7 |
| CYCS | cytochrome c, somatic |
| ELANE | elastase, neutrophil expressed |
| GPX4 | glutathione peroxidase 4 |
| GSDMA | gasdermin A |
| GSDMB | gasdermin B |
| GSDMC | gasdermin C |
| GSDMD | gasdermin D |
| GSDME | gasdermin E |
| GZMB | granzyme B |
| HMGB1 | high mobility group box 1 |
| IL18 | interleukin 18 |
| IL1A | interleukin 1 alpha |
| IL1B | interleukin 1 beta |
| IL6 | interleukin 6 |
| IRF1 | interferon regulatory factor 1 |
| IRF2 | interferon regulatory factor 2 |
| NLRC4 | NLR family CARD domain containing 4 |
| NLRP1 | NLR family pyrin domain containing 1 |
| NLRP2 | NLR family pyrin domain containing 2 |
| NLRP3 | NLR family pyrin domain containing 3 |
| NLRP6 | NLR family pyrin domain containing 6 |
| NLRP7 | NLR family pyrin domain containing 7 |
| NOD1 | nucleotide binding oligomerization domain containing 1 |
| NOD2 | nucleotide binding oligomerization domain containing 2 |
| PJVK | pejvakin |
| PLCG1 | phospholipase C gamma 1 |
| PRKACA | protein kinase cAMP-activated catalytic subunit alpha |
| PYCARD | PYD and CARD domain containing |
| SCAF11 | SR-related CTD associated factor 11 |
| TIRAP | TIR domain containing adaptor protein |
| TNF | tumor necrosis factor |
| TP53 | tumor protein p53 |
| TP63 | tumor protein p63 |

**Table S2. Pyroptosis related genes difference.**

| **Gene** | **conMean** | **treatMean** | **logFC** | **pValue** | **fdr** |
| --- | --- | --- | --- | --- | --- |
| GSDMC | 0.018588 | 0.165932 | 3.158149 | 1.06E-18 | 2.53E-17 |
| ELANE | 0.787419 | 0.07102 | -3.47083 | 1.56E-26 | 7.50E-25 |
| NLRP7 | 0.203629 | 0.055458 | -1.87647 | 7.86E-16 | 6.29E-15 |
| CASP5 | 16.03188 | 3.945627 | -2.02262 | 1.50E-17 | 1.80E-16 |
| IL1A | 0.16444 | 1.575974 | 3.260613 | 2.22E-15 | 1.46E-14 |
| NOD2 | 0.452895 | 1.33387 | 1.558369 | 7.01E-12 | 2.80E-11 |
| GZMB | 2.26311 | 10.81795 | 2.257049 | 1.56E-11 | 5.22E-11 |
| GSDMA | 0.325645 | 1.899486 | 2.544236 | 5.20E-15 | 2.77E-14 |
| IL1B | 2.99772 | 10.41135 | 1.79622 | 4.84E-07 | 8.61E-07 |
| PLCG1 | 4.582236 | 10.13129 | 1.144694 | 2.43E-15 | 1.46E-14 |

**Table S3. Pyroptosis related lncRNA geneCoef.**

| **Gene** | **Coef** |
| --- | --- |
| SNHG26 | 0.6661529 |
| MYOSLID | 2.8228213 |
| TMEM147-AS1 | 0.02900612 |
| CCDC183-AS1 | 0.0793203 |
| AL354836.1 | 0.06699034 |
| LINC00174 | 0.07248961 |
| AC023157.2 | 0.36800457 |
| AC084125.2 | 0.21302489 |
| AL137782.1 | -0.540688 |

**Table S4. Immune correlation result.**

| **Immune** | **cor** | **pvalue** |
| --- | --- | --- |
| T cell CD4+_TIMER | 0.281507154 | 1.70E-09 |
| Neutrophil_TIMER | 0.191421624 | 4.88E-05 |
| Macrophage_TIMER | 0.187156756 | 6.99E-05 |
| Myeloid dendritic cell_TIMER | 0.191387673 | 4.89E-05 |
| B cell memory_CIBERSORT | 0.106050963 | 0.025110803 |
| T cell CD4+ memory resting_CIBERSORT | -0.126474719 | 0.007490015 |
| Monocyte_CIBERSORT | 0.13129863 | 0.005484489 |
| Myeloid dendritic cell resting_CIBERSORT | -0.131089952 | 0.005560085 |
| Mast cell resting_CIBERSORT | -0.130794673 | 0.005668655 |
| B cell memory_CIBERSORT-ABS | 0.108360439 | 0.022093655 |
| T cell CD8+_CIBERSORT-ABS | 0.14814135 | 0.001706273 |
| T cell follicular helper_CIBERSORT-ABS | 0.179318034 | 0.000140525 |
| T cell regulatory (Tregs)_CIBERSORT-ABS | 0.117945589 | 0.012682071 |
| NK cell activated_CIBERSORT-ABS | 0.093304488 | 0.048925707 |
| Monocyte_CIBERSORT-ABS | 0.156323434 | 0.000924755 |
| Macrophage M0_CIBERSORT-ABS | 0.22774602 | 1.17E-06 |
| Macrophage M1_CIBERSORT-ABS | 0.147347571 | 0.001807864 |
| Macrophage M2_CIBERSORT-ABS | 0.207855268 | 9.61E-06 |
| Myeloid dendritic cell resting_CIBERSORT-ABS | -0.10509763 | 0.026456284 |
| B cell_QUANTISEQ | 0.227361425 | 1.22E-06 |
| Macrophage M2_QUANTISEQ | 0.145543137 | 0.002059732 |
| T cell CD8+_QUANTISEQ | 0.117029196 | 0.013395661 |
| T cell regulatory (Tregs)_QUANTISEQ | 0.161056834 | 0.000639974 |
| uncharacterized cell_QUANTISEQ | -0.101673439 | 0.031850781 |
| T cell_MCPCOUNTER | 0.238759192 | 3.65E-07 |
| cytotoxicity score_MCPCOUNTER | 0.137604689 | 0.003619156 |
| NK cell_MCPCOUNTER | 0.174463978 | 0.000217985 |
| B cell_MCPCOUNTER | 0.13759211 | 0.00362226 |
| Monocyte_MCPCOUNTER | 0.241122912 | 2.78E-07 |
| Macrophage/Monocyte_MCPCOUNTER | 0.241122912 | 2.78E-07 |
| Myeloid dendritic cell_MCPCOUNTER | 0.13388834 | 0.004648296 |
| Neutrophil_MCPCOUNTER | 0.144126933 | 0.002299318 |
| Endothelial cell_MCPCOUNTER | 0.236264403 | 4.85E-07 |
| Cancer associated fibroblast_MCPCOUNTER | 0.266299405 | 1.28E-08 |
| Myeloid dendritic cell activated_XCELL | 0.18716805 | 6.98E-05 |
| T cell CD4+ memory_XCELL | -0.104150256 | 0.027854393 |
| T cell CD4+ naive_XCELL | 0.193556891 | 3.87E-05 |
| T cell CD4+ central memory_XCELL | 0.128938794 | 0.006395751 |
| Common lymphoid progenitor_XCELL | -0.163899942 | 0.000518691 |
| Myeloid dendritic cell_XCELL | 0.128496156 | 0.006581072 |
| Endothelial cell_XCELL | 0.171679532 | 0.000269945 |
| Hematopoietic stem cell_XCELL | 0.117698721 | 0.012870928 |
| Macrophage_XCELL | 0.09289501 | 0.049930532 |
| Macrophage M1_XCELL | 0.130252125 | 0.005873119 |
| Monocyte_XCELL | 0.098288653 | 0.037992925 |
| B cell naive_XCELL | 0.12049073 | 0.010873045 |
| T cell gamma delta_XCELL | -0.153406433 | 0.001154409 |
| immune score_XCELL | 0.168376605 | 0.000361436 |
| stroma score_XCELL | 0.170172636 | 0.000306071 |
| microenvironment score_XCELL | 0.200880968 | 2.00E-05 |
| Cancer associated fibroblast_EPIC | 0.283122058 | 1.36E-09 |
| T cell CD4+_EPIC | 0.110101809 | 0.020072342 |
| T cell CD8+_EPIC | -0.105625146 | 0.025744317 |
| Endothelial cell_EPIC | 0.210180836 | 7.96E-06 |
| Macrophage_EPIC | 0.227140511 | 1.34E-06 |
| NK cell_EPIC | 0.182526597 | 0.000108725 |
| uncharacterized cell_EPIC | -0.323052628 | 3.59E-12 |
